# Development and Evaluation of a Nanoparticle-Based Immunoassay for Rotavirus Detection: A Suitable Alternative to ELISA and PCR in Low-Income Setting

**DOI:** 10.3390/mps8040081

**Published:** 2025-07-17

**Authors:** Margaret Oluwatoyin Japhet, Adeogo Timilehin Bankole, Temiloluwa Ifeoluwa Omotade, Oyelola Eyinade Adeoye, Oladiran Famurewa, Simeon K. Adesina

**Affiliations:** 1 Department of Microbiology, Faculty of Science, Obafemi Awolowo University, Ile-Ife 220103, Osun State, Nigeria; 2 Department of Pharmaceutical Sciences, College of Pharmacy, Howard University, Washington, DC 20059, USA; 3 Department of Community Medicine, Osun State University, Osogbo 210001, Osun State, Nigeria; 4 Department of Microbiology, Faculty of Science, Ekiti State University, Ado-Ekiti 362103, Ekiti State, Nigeria

**Keywords:** diarrhoea, rotavirus, diagnosis, immunoassay development, children, ELISA, PCR

## Abstract

Every year, diarrhoea is responsible for >1 million deaths in children with ages from 0 to 5 years, with rotavirus as the leading cause. The regions most affected lack routine rotavirus diagnosis due to high cost, lack of necessary equipment and shortage of trained-personnel for Enzyme-Link-Immunosorbent-Assay (ELISA) and molecular methods. We report the development and evaluation of a cheap, nanoparticle-based immunoassay for routine machine-free rotavirus diagnosis. In this work, optimal conditions for oxidation of cotton swabs and aldehyde production for kit development was confirmed by Fourier-Transform Infrared Spectroscopy (FTIR). Lactoferrin (LF) needed to bind the virus to the cotton swab was immobilised on activated cotton swabs, followed by the capture of commercial rotavirus antigen on LF-immobilised swabs. This was dipped in coloured nanobeads covalently coupled to rotavirus-group-specific monoclonal antibody for visual rotavirus detection. Subsequently, rotavirus detection by nanoassay, commercial ELISA and quantitative reverse transcription PCR were compared using same set of 186 stool samples and subjected to statistical analyses. Optimal oxidisation condition was observed using 48 mg/mL NaIO_4_ in 0.1 M sodium acetate buffer at 35 °C for 9 h. Rotavirus detection was confirmed visually by blue colour retention on swabs after several washings. Sensitivity, specificity, positive-predictive-value and negative-predictive-value of ELISA in rotavirus detection were 60%, 84%, 53% and 88%, respectively, while our immunoassay showed performance at 88%, 94%, 82% and 96%. This immunoassay will provide effective rotavirus public health interventions in low-and-middle-income countries with high morbidity/mortality.

## 1. Introduction

Diarrhoeal diseases claim more than 1 million lives annually and are a leading cause of death in children younger than 5 years [1]. In 2021, an estimated 59 million (47.2–73.2) disability-adjusted life-years (DALYs) were attributable to diarrhoeal diseases globally, with 30.9 million (23.1–42.0) of these affecting children younger than 5 years [1].

In developing countries, it has been estimated that 1.8 million people die annually due to diarrheal diseases and more than 80% of them are children aged under five years [2,3]. Diarrhoea is responsible for the death of more than 90% of children under-five years of age in low- and middle-income countries. In 2021, the number of estimated global deaths from diarrhoea in children younger than 5 years was 444,0000, with LMICs accounting for more than 99% of these deaths [4]. In Nigeria, diarrhoea is the second most common cause of paediatric death, representing 18.8% of all annual paediatric deaths. Yearly, this is estimated to be about 150,000 lives in the country [5].

Diarrhoea is caused by a wide range of aetiological agents including viruses, bacteria and parasites, but viral agents are the major aetiology of diarrheal disease in Africa [6,7,8,9]. Viral gastroenteritis is a common cause of morbidity and mortality worldwide [10]. Group A rotavirus (RVA) is the most common cause of severe diarrhoea and deaths in infants and young children worldwide [1,4,11,12,13,14]. Indiscriminate use of antibiotics in diarrhoea management has been reported, despite there being no obvious diagnostic nor epidemiological basis that the children were suffering from infections for which antibiotics use is appropriate [15,16]. It has been reported that the rapid diagnosis of rotavirus infections in patients admitted to the hospital with symptoms of gastroenteritis would enable better treatment of the patient. Additionally, accurate diagnosis of rotavirus is essential since it obviates the unnecessary use of antibiotic therapy [17]. In developing countries, the diagnosis of diarrhoeal infections relies mainly on clinical symptoms while laboratory diagnosis of gastrointestinal infections (especially of viral origin) is largely neglected [18] compared to the developed countries where laboratories increasingly apply real time PCR assays to diagnose gastrointestinal infections.

Various studies have reported the detection of different diarrhoeal viruses using different methods, ranging from electron microscopy, latex agglutination (LA) assays, immunochromatography, ELISA, and more recently, molecular methods such as quantitative or semi-quantitative PCR [19,20,21,22]. However, these methods have their shortcomings. For instance, the latex agglutination (LA) assays for rotavirus diagnosis are simple, fast, require no special equipment or professionals, but have low sensitivity [23] and may not detect low titres of rotavirus (RV), thereby resulting in false negative and indeterminate results [24].

Although enzyme-linked immunosorbent assay (ELISA) enables the simultaneous analysis of multiple clinical samples, it typically requires a long turnaround time (several hours), a complex detection system to read through the results is necessary and its diagnostic accuracy is relatively small compared to molecular methods [25]. It is highly possible that uninfected individuals can be inadvertently exposed to pathogens and become infected while waiting for test results, especially where samples have to be pooled for days, in order to run the plate once, making conventional ELISAs not suitable for point of care (POC) detection [25].

The use of polymerase chain reaction (PCR) techniques for virus detection and quantification offers the advantages of high sensitivity and reproducibility, combined with an extremely broad dynamic range. The establishment of PCR-based detection methods has provided the basis for reliable detection of viral nucleic acids in the clinical setting [26]. The use of the polymerase chain reaction (PCR) in molecular diagnostics has increased to the point where it is now accepted as the gold standard for detecting nucleic acids from a number of origins and it has become an essential tool in the research laboratory [27]. Although RT-PCR is the gold standard for diagnosis, its long operation time and requirement for highly purified samples, specialised instruments and trained professionals has limited its clinical application [27]. Hence routine diagnosis using PCR is currently not feasible in developing countries, including Nigeria, due to the high cost, unavailable equipment and lack of trained personnel. There is, therefore, the need for a cheap, rapid, easy to perform and equipment free rotavirus diagnostic assay for routine detection of rotavirus in endemic regions which are mostly low-income countries.

In a quest for improved diagnostic and therapeutic techniques, harnessing the potentials of nanomaterials, has recently taken the stage in scientific research [28,29]. Nano-diagnostics involves the integration of nanotechnology and its principles for diagnostic applications, providing new and simple methods for evaluating patients’ samples and identifying disease biomarkers at an early stage with enhanced sensitivity and specificity [30].

Cotton (cellulose) swabs are widely applied for recovering pathogens from contaminated surfaces and have been explored for both sample collection and as a supporting matrix for sensors in bacterial and viral detection [31,32,33,34]. In this paper, we explore the development of a rotavirus immunoassay, using cotton swab in a nanoparticle-based immunoassay method. Subsequently, we evaluated the performance of the assay by analysing the same set of diarrhoeic and control stool samples, using molecular method (quantitative PCR), enzyme-linked immunosorbent assay (ELISA) and our newly developed nanoparticle-based immunoassay. The goal is to determine the suitability of our newly developed nanoparticle-based immunoassay for routine diagnosis of RVA in children with diarrhoea. The use of this assay will improve diarrhoea management in developing countries and reduce indiscriminate use of antibiotics.

## 2. Materials and Methods

### 2.1. Ethical Approval

Permission to carry out this study was obtained from the Ethics and Research Committee (ERC) of the Obafemi Awolowo University Teaching Hospital Complex, Ile-Ife, Osun State, in compliance with established research ethics protocols (protocol number ERC/2021/04/12).

### 2.2. Study Design, Location and Sample Characteristics

This is a cross-sectional study carried out from June 2021 to June 2022 to obtain diarrhoea incidence for one year. Patients were recruited from a tertiary health hospital (Obafemi Awolowo University Teaching Hospital—OAUTHC) and two primary health care centres—Urban Comprehensive Health Centre, Eleyele and Enuowa State Hospital, all in Osun State, Southwestern part of Nigeria. A total of 186 participants (Male 101; Female 85; age 0–5 years), comprising 121 children with diarrhoea and 65 children without diarrhoea (controls) were enrolled in the study. Controls are defined as children without diarrhoea in the last 14 days prior sample collection.

#### 2.2.1. Inclusion Criteria

Children (0–5 years) presenting with diarrhoea as outpatient or for admission at any of the three health care facilities whose parent(s) or guardian(s) consent to participate in the study were recruited for this study. The case definition of diarrhoea is the passage of three or more liquid or loose stool within a 24 h duration with or without vomiting, fever or dehydration. Also, children brought to the hospital for immunisation or routine visits within the age range but without record of diarrhoea for at least preceding 14 days were recruited as control.

#### 2.2.2. Exclusion Criteria

Children older than five years or those within the inclusion criteria whose parents do not consent to be part of the study were excluded from the study.

### 2.3. Sample Collection

Stool samples were collected into plain pre-labelled sterile sample bottles. The bottles were labelled with laboratory identity numbers, unique to each child and the numbers were dissociated from individual details to ensure confidentiality. The collected stool samples were transported on ice packs to the Virology and Molecular Research Laboratory, Department of Microbiology, Obafemi Awolowo University, Ile-Ife, where samples were stored in aliquots at −80 °C until further processing.

### 2.4. Rotavirus Assay Development

#### 2.4.1. Materials for Assay Development

Sodium periodate (NaIO_4_), sulfuric acid, phosphate-buffered saline (PBS), Bovine serum albumin (BSA), 1-ethyl-3-(3-dimethylaminopropy)carbodiimide hydrochloride (EDC), Lactoferrin from bovine milk, sodium acetate, acetic acid and N-hydroxy succinimide (NHS) were purchased from Sigma-Aldrich (St. Louis, MO, USA). Cotton swabs and nanobeads containing carboxylic acid functional groups were purchased from Harmony Lab & Safety Supplies (Garden Grove, CA, USA) and Bangs Laboratories Inc. (Fishers, IN, USA), respectively. Rotavirus antigen and Rotavirus A (RVA) VP6 monoclonal antibody were purchased from ATCC (American Type Culture Collection) (Manassas, VA, USA) and Life Technologies, Thermofisher Scientific (San Jose, CA, USA), respectively.

#### 2.4.2. Optimisation of Periodate Oxidisation of Cotton Swabs

To evaluate the effect of reaction conditions such as concentration of sodium periodate (NaIO_4_), reaction temperature, and time of reaction on degree of oxidation of the cotton on a solid support (cotton swab) to yield reactive aldehyde intermediates, optimisation of each reaction condition was carried out. Cotton swabs were oxidised using varying concentrations (12 mg/mL, 24 mg/mL, 36 mg/mL and 48 mg/mL) of NaIO_4_ in 0.1 M sodium acetate buffer (pH 5.2) according to published methods [31,35] with slight modifications. Cotton swabs were immersed in the solutions and incubated with shaking, using a Fisherbrand laboratory shaker capable of 360° rotation, at different reaction times (3 h, 6 h, 9 h, 12 h and 24 h) under varying temperature (25 °C, 35 °C and 50 °C) conditions, to ascertain optimal oxidation conditions. Reports have shown that the concentration of free aldehyde groups on cotton oxidised using periodate is greatly affected by moisture which makes it difficult to detect the aldehyde product of periodate oxidation using IR spectroscopy [36]. Hence, after oxidation, cotton swabs were washed thoroughly with ice-cold distilled water and dried in a vacuum oven overnight. The conversion of the dihydroxyl groups of cellulose to aldehyde in our experiment was confirmed using FTIR. A characteristic carbonyl stretch at around 1720–1740 cm^−1^ confirms oxidation and conversion to aldehyde and optimal conditions were selected for kit development.

#### 2.4.3. Immobilisation of Lactoferrin on Oxidised Cotton Swab

Rotavirus antigen cannot bind to the activated cotton swabs, hence the need for the activated cotton swab to be bound to Lactoferrin (LF), a globular glycoprotein which is known to bind cells [35,37,38], which can then bind rotavirus antigen. LF was first immobilised on the activated cotton by the reaction of amino groups on LF molecules with aldehyde groups on the activated cotton swab. Activated cotton swabs were immersed in 20 mL lactoferrin solution (1 mL, 200 µg/mL of lactoferrin in 19 mL PBS) at 4 °C overnight on a laboratory shaker capable of 360° rotation at 10 rpm. Once completed, the LF-conjugated cotton swabs were washed extensively with PBS to remove unbound lactoferrin. Unreacted active aldehyde groups on the swab were blocked by incubating the LF-conjugated cotton swabs in 1 mg/mL of bovine serum albumin (BSA) at 4 °C on a shaker for 30 min. The swabs were thoroughly washed thereafter and stored in PBS at 4 °C till further use. Similarly, control swabs were prepared by using BSA (1 mg/mL; 20 mL) in place of lactoferrin and reacted overnight by incubation at 4 °C on the shaker.

#### 2.4.4. Activation of Nanobeads and Conjugation of RVA-VP6 Monoclonal Antibody on Carboxyl-Functionalized Coloured Nanobeads

Beads were prepared for conjugation according to the manufacturer’s protocol (Bangs Laboratories, Inc. Fishers, IN, USA, TechNote 205) with slight modifications. Briefly a suspension of nanobeads (2 mL of 50 mg/mL beads supplied as 10% solid) was pelleted by centrifugation (20,000 rpm at 15 degrees for 5 min) to recover the nanobeads. The pelleted beads were washed by resuspending in activation buffer (MES; pH 5.7, 10 mL) and vortexed to redisperse, followed by centrifugation using the same conditions above. This was repeated. For antibody conjugation to beads, 100 mg of EDC was added to the beads and shaken for 10 min on a laboratory shaker, followed by addition of NHS (60 mg; 0.000522 mol) to ensure formation of a stable NHS ester intermediate. The reaction was allowed to continue for another 30 min on the laboratory shaker at room temperature. The beads were then washed twice in coupling buffer by sequential vortexing and centrifugation steps to remove excess coupling agent and resuspended in 5 mL of coupling buffer to achieve a homogenous suspension.

The rotavirus Group A (RVA) VP6 monoclonal antibody purchased from Life Technologies, Thermofisher Scientific (MA516297) was used for the conjugation. Antibody conjugation was performed according to the manufacturer’s protocol (Bangs Laboratories, Inc TechNote 205) with slight modifications. The amount of antibody needed for conjugation was calculated using the following formula:

S = (6/ρSd)(C)

where

S = amount of protein required to achieve surface saturation (mg protein/g of microspheres),

ρS = density of solid sphere (g/cm^3^),

d = mean diameter (µm),

C = capacity of microsphere surface for a given protein (mg protein/mL of sphere surface).

The protein (RVA VP6 monoclonal antibody) was dissolved in 5 mL coupling buffer and added to the activated nanobead suspension. The reaction was allowed to continue for 3 h at room temperature on a laboratory shaker. Upon completion of the reaction, the antibody conjugated nanobead suspension was washed twice in coupling buffer by sequential vortexing and centrifugation steps to remove unreacted antibody and resuspended in 10 mL of quenching solution (hydroxylamine 40 mM/L and 1% BSA), on a laboratory shaker for 30 min. This was followed by washing with 10 mL PBS and resuspension in 5 mL storage buffer. The antibody conjugated nanobead suspension was kept at 4 °C until used.

#### 2.4.5. Detection of Rotavirus as Proof of Concept

Oxidised-lactoferrin-coupled swab was used to obtain RV antigen and dipped in Ab coupled nanobeads for RV detection. As a proof of concept, rotavirus antigen culture stock (VR-2272 Rotavirus Group A Strain) purchased from ATCC was used to determine the performance of the developed kit in rotavirus detection. A total of 1000 µL RVA antigen was diluted with 1 mL PBS. For adhesion or adsorption of rotavirus antigen (RV Ag) to the swab, the cotton-immobilised lactoferrin swab was dipped in RVA antigen solution for 10 min, to facilitate LF binding to RV on the swab. The cotton-LF-RVA Ag obtained was washed severally with PBS to remove unbound antigen and subsequently immersed in rotavirus VP6 antibody coupled bead suspension for 10 min to facilitate binding to antibody-coupled coloured nanobeads. The swab was washed severally with PBS to remove unbound beads. Coupling of the antibody-bound coloured beads to swab was visually evaluated for blue colour.A patent application was submitted for the developed assay.

### 2.5. Sample Evaluation by ELISA, Newly Developed RV Nanoparticle Immunoassay and Molecular Methods

The 186 stool samples collected from 121 diarrhoeic and 65 control children were screened for rotavirus using commercial ELISA, the developed nanoparticle-based immunoassay and quantitative reverse transcription polymerase chain reaction (as gold standard).

#### 2.5.1. Stool Preparation

Ten percent faecal suspension of the samples were first prepared using 1 g of formed stool or 100 µL of watery stool in 1000 µL of phosphate-buffer saline [PBS] [39].

#### 2.5.2. Rotavirus Detection by ELISA

Rotavirus antigen detection in the collected samples was performed using the Enzyme-Linked Immunosorbent Assay (ELISA) technique, with the Human Rotavirus Antigen (RV-Ag) ELISA Kit (Melsin, Changchun, China), adhering strictly to the protocol outlined by the manufacturer.

Briefly, a total volume of 50 µL from the positive and negative controls were dispensed separately to the designated wells. Afterwards, 40 µL of the PBS diluted faecal solutions were pipetted into the testing wells, excluding the blank well, and each testing well containing a test sample was supplied with 10 µL of sample diluent. Subsequently, a volume of 100 microliters (µL) of Horseradish Peroxidase (HRP)-conjugated reagent was accurately dispensed into each well and gently tapped to give room for proper mixing before it was incubated at 37 °C for 60 min. After incubation, plate was well aspirated and washed repeatedly for 5 times by filling each well with 350 µL of wash solution. Thereafter, equal volumes of 50 microliters (µL) of Chromogen Solution A and Chromogen Solution B were accurately dispensed with a protection from light and then incubated at 37 °C for 15 min. Finally, the chromogenic reaction was stopped by adding 50 µL of stop solution to each well, and the resulting optical density values were then quantified using an ELISA microplate reader at 450 nm wavelength within 15 min.

#### 2.5.3. Rotavirus Detection Using Our Developed RV Nanoparticle-Based Immunoassay

Detection of rotavirus antigen from collected stool samples was carried out using the newly developed RV immunoassay following the proof-of-concept guidelines.

##### Principle of the Test

The qualitative nanoparticle-based immunoassay diagnostic method helps detect rotavirus Antigen (RV-Ag) in biological samples. Immobilised lactoferrin on activated cotton swabs captures rotavirus antigen available in stool sample. A wash step removes any unbound RV-Ag. Covalently coupled VP6 monoclonal antibody on blue-coloured nanobeads bind rotavirus on the cotton swab when dipped in the solution. Retention of the blue colour of the nanobeads after several washing confirms the presence of human RV antigen in stool sample. The intensity of the colour is directly proportional to viral concentration in the stool, while the blue colour is washed off in the rotavirus negative samples.

##### Test Procedure

From 10% faecal suspension of stool samples, 100 µL were dispensed into labelled collection tubes. Thereafter, lactoferrin-bound cotton swabs were suspended in each sample tubes for 10 min and swirled at intervals for a proper mix. Each cotton swab was rinsed severally in separately labelled Eppendorf tubes filled with 1.5 mL of Phosphate-Buffer Saline (PBS) in the 2 mL tube. Subsequently, five hundred microliter (500 µL) of rotavirus VP6 antibody coupled beads were dispensed into newly labelled Eppendorf tubes and the rinsed cotton swabs were placed in the liquid for 10 min. After 10 min, the wash step in Eppendorf tubes was carried out and the swabs were placed on a white background and the results read visually.

#### 2.5.4. Rotavirus Detection by Real-Time Quantitative Reverse Transcription PCR (qRT-PCR)

##### Viral RNA Extraction

Viral RNA was extracted from stool samples using the QIAamp^®^ Viral RNA extraction kit (QIAgen, Hilden, Germany) following manufacturer’s protocols and about 60 µL purified RNA obtained from each stool sample were stored at −80 °C till further analysis. Denaturation at 95 °C for 1 min followed by chilling immediately on ice for about 2 min was performed on 2.2 μL RNase-free water and 2.6 μL RNA template mixture. Reverse transcription and quantitative real time polymerase chain reaction (qRT-PCR) specific for NSP4 of RVA was then carried out in a single-step approach as previously described [39] with slight modifications. Briefly, Luna Universal Probe One-Step QRT-PCR Kit (New England Biolabs^®^ Inc. Ipswich, MA, USA) was used in PCR tubes according to manufacturer’s instruction, hence 16 µL of the master mix with 4 µL template from the denaturation product were used, to make a 20 µL final volume per sample reaction instead. The master mix contained Nuclease free water, Universal Probe One-Step Reaction mix, WarmStart RT enzyme mix, RVA forward primer (5′-GCTTTTAAAAGTTCTGTTCCGAG) and RVA reverse primer (5′-ACTCAATGTGTAGTTGAGGTCGG) and a VIC-conjugated, minor groove binder (MGB) probe (5′-VIC-ATCTTTCCGCACGC-3MGBNFQ). The PCR tubes were centrifuged and placed in a thermal cycler. Rotavirus RNA detection was accomplished using the Applied Biosystems QuantStudio™ 5 Real-Time PCR System, which facilitated the amplification and fluorescence-based detection under the following precisely controlled thermal cycling conditions: 50 °C for 15 min, 95 °C for 2 min, 45 cycles (94 °C for 15 s, 55 °C for 32 s).

### 2.6. Data Analysis

Statistical analyses were performed by Fischer’s exact tests, using two-tailed significance. SPSS version 25.0.1 for Windows and graph pad prism 5.0. were used to determine the predictive values of the selected parameters [sensitivity (SS), specificity (SP), negative predictive value (NPV), positive predictive value (PPV)], using PCR as the gold standard. A *p*-value < 0.05 was considered statistically significant.

## 3. Results

### 3.1. Optimal Oxidation Conditions for Cotton Swabs

#### Effect of Periodate Concentration

For the initial experiments conducted to evaluate the effect of reaction temperature on the oxidation reaction, our results indicated that the oxidation reaction progressed best at 35 °C; thus, all oxidation reactions were performed at 35 °C. For the four different concentrations (12 mg/mL, 24 mg/mL, 36 mg/mL and 48 mg/mL) of periodate used for cotton swab oxidation at different reaction time periods, the FTIR spectra obtained (Supplementary Data S1–S4), showed either no oxidation (Supplementary Data S1a–c, S2a,b, S3a,b and S4b–d) or varying oxidation peaks (Supplementary Data S1d, S2c,d, S3c,d and S4a). Oxidisation of cotton swabs with 48 mg/mL NaIO_4_ in 0.1 M sodium acetate buffer at 35 °C for 9 h was observed to be the optimal oxidation condition for synthesis of the aldehyde as well as preservation of the physical structure of cotton swab; hence, these conditions were selected for assay development.

### 3.2. Lactoferrin Immobilisation on Oxidised Cotton Swab

Lactoferrin immobilisation on the cotton swab was successfully carried out after successful conversion of alcohol groups to the dialdehyde as characterised by FTIR. Lactoferrin immobilisation on the cotton swab was confirmed by the disappearance of the aldehyde peak on the oxidised cotton swab after reaction with lactoferrin (Supplementary Data S5), suggesting the conjugation of the amino groups on lactoferrin to the synthesised reactive aldehyde groups on the swabs via an imine (Schiff base) formation reaction [40,41]. The lactoferrin conjugated cotton swabs were kept at 4 °C until used.

### 3.3. Experimental Setup and Detection of Rotavirus as Proof of Concept

**Figure 1:**
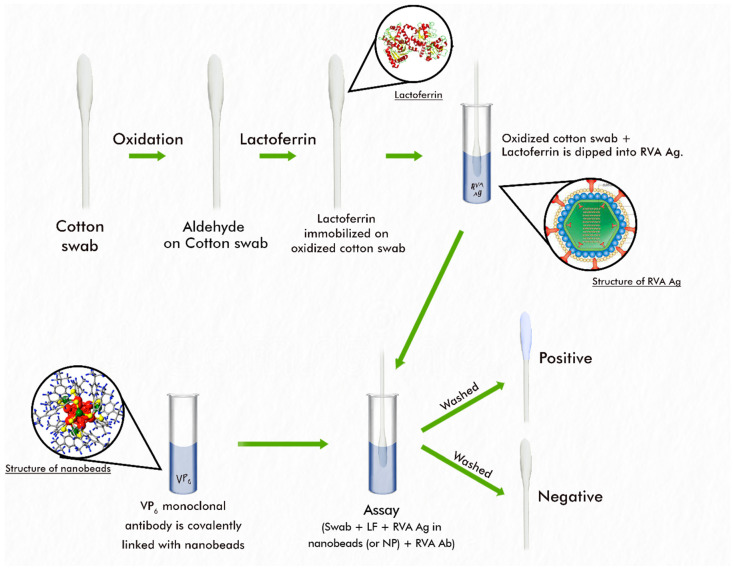
Experimental setup for the development and testing of the rotavirus nanoparticle-based immunoassay.

Details of the experimental setup for the development and testing of the rotavirus nanoparticle immunoassay development procedure are described in Figure 1.

**Figure 2:**
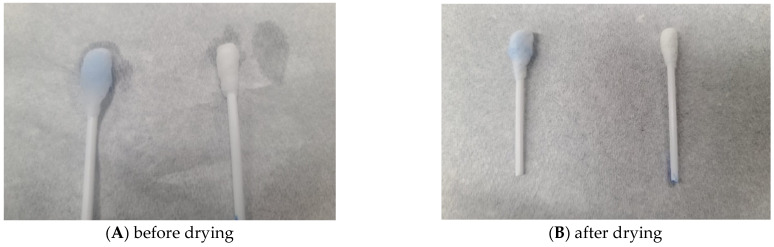
Rotavirus positive swab (left) and negative (right). (**A**) before drying (**B**) after drying.

Successful binding of rotavirus antigen to lactoferrin, making the RV Ag available for binding to RV Ab on the blue-coloured beads and hence detection, was confirmed by the retention of the blue colour on cotton swabs despite multiple washings (Figure 2A left). For control swabs, the colour was washed off, indicating that no RVA antigen was attached to the swab due to the absence of lactoferrin (Figure 2A right). Figure 2B shows the positive and negative cotton swabs after oven drying. The Patent Registration Certificate (NP/P/2023/88) is available on request.

### 3.4. Rotavirus Detection Using PCR, ELISA and Nanoparticle Developed Kit

For the same set of 186 samples (121 diarrhoeic and 65 control children) tested, RVA detection rate by ELISA, developed nanoparticle assay and qRT-PCR were determined as follows.

#### 3.4.1. Detection of Rotavirus from Stool Samples Using ELISA

**Figure 3:**
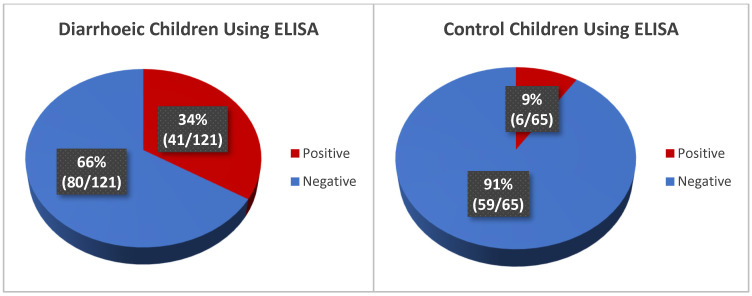
Rotavirus positivity of diarrhoea and control group using ELISA.

Using ELISA, rotavirus antigen detection among diarrhoeic children shows a high rotavirus infection of 34% (41/121) while 9% (6/65) of the control group were rotavirus positive (Figure 3).

#### 3.4.2. Detection of Rotavirus Using the Developed RV Nanoparticle-Based Immunoassay

**Figure 4:**
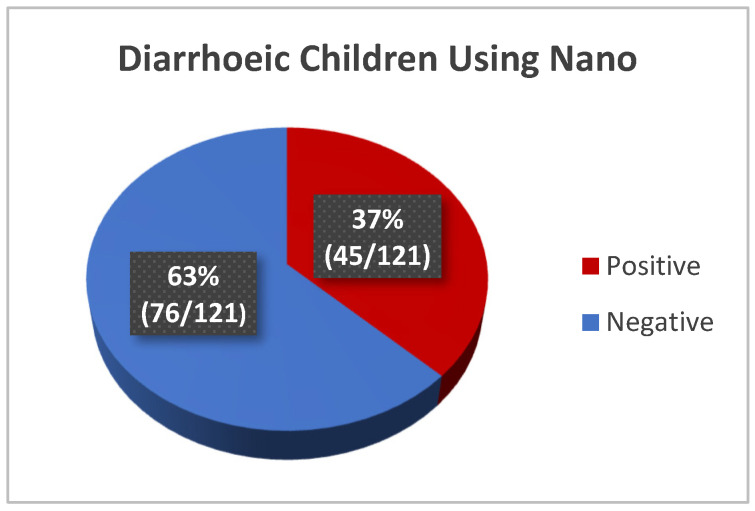
Rotavirus Positivity of Diarrhoea Group using Nanoparticle-based immunoassay.

**Figure 5:**
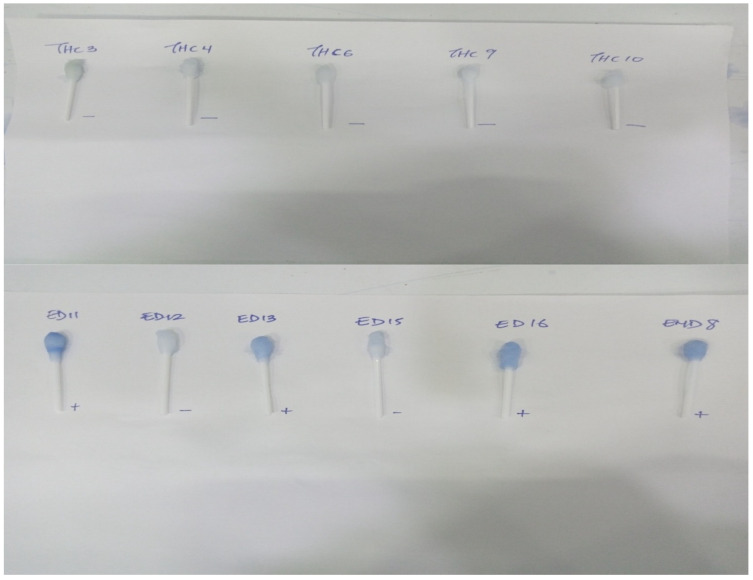
Representative visual reading of RV detection using the developed nanoparticle-based immunoassay in diarrhoeic and control samples. ED represents diarrhoea samples from Urban Comprehensive Health Center, Eleyele, THC represents control samples from OAUTHC.

**Figure 6:**
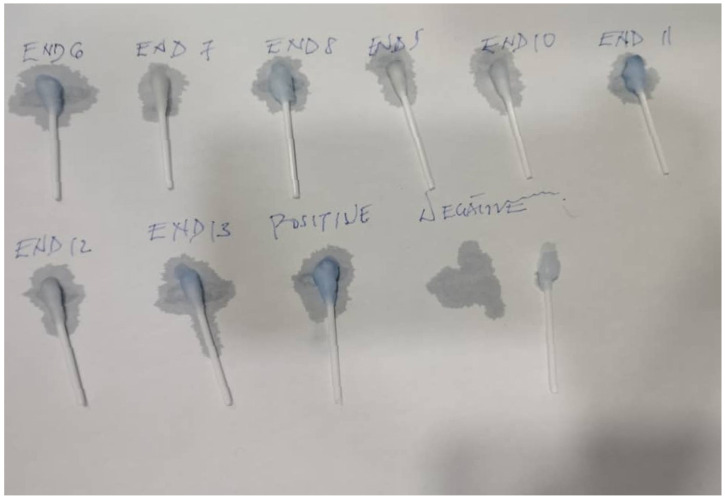
Validation of Nanoparticle kit with ELISA Positive and Negative Controls. END represents diarrhoea Samples from Enuowa Primary Health Center.

The nanoparticle kit detected a high prevalence of rotavirus infection of 37% (45/121) in the diarrhoeic children while none of the 65 control samples were found positive for rotavirus (Figure 4). Representative visual reading of diarrhoeic and control samples is shown in Figure 5. To further validate the test, the positive and negative controls in the ELISA kit, alongside some diarrhoeic samples were also tested with the nanoparticle kit and ELISA controls were confirmed positive and Negative, respectively, by the kit (Figure 6)

#### 3.4.3. Detection of Rotavirus in Diarrhoeic Children Using Molecular Method (qRT-PCR)

**Figure 7:**
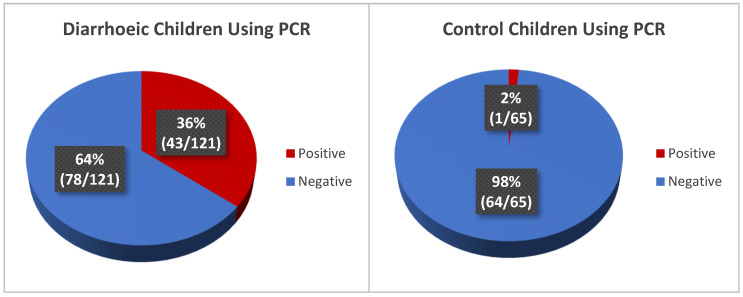
Rotavirus A positivity in diarrhoeic and control group using molecular method.

The diarrhoeic stool samples and control samples were evaluated for rotavirus infection using reverse transcription real-time polymerase chain reaction method (qRT-PCR). The detection of Rotavirus among the diarrhoeic and control children shows a prevalence of 36% (43/121) and 2% (1/65), respectively (Figure 7).

#### 3.4.4. Comparison of RV Detection by ELISA, the Developed Nanoparticle-Based Immunoassay and Quantitative PCR

Table 1 shows the rate of RV detection by the 3 methods in the diarrhoeic and control group while Table 2a,b shows the true positives and negatives for ELISA and nanokit assay in comparison with PCR. The SS, SP, PPV and NPP for the same set of 186 samples (121 diarrhoeic and 65 control) samples for ELISA in detecting rotaviral infection were found to be 60%, 84%, 53% and 88%, respectively, while for our developed immunoassay, the values were 88%, 94%, 82% and 96%, respectively, showing that our kit performed better than the ELISA (Table 3).

## 4. Discussion

This study reports the development of a nanoparticle-based rotavirus immunoassay and its suitability as a cheaper alternative to molecular methods in the routine detection of rotavirus (RV) in low- and middle-income countries (LMIC) where cost and other factors hinder routine rotavirus diagnosis. This will improve diarrhoea management, since reports have shown that optimum management of childhood diarrhoea in low-resource settings has been hampered by insufficient data on aetiology, among other factors [42].

In recent years, nanotechnologies have been applied to human health with promising results [43]. In this work, we report the oxidation of cotton swabs with NaIO_4_ solutions under different conditions, to obtain a suitable platform for lactoferrin immobilisation in the development of a rapid, simple, cheap and machine free rotavirus diagnostic immunoassay. Several publications have reported NaIO_4_ oxidation of cellulose to produce aldehyde [44,45,46,47,48]. Although, oxidation of cellulose with periodate is a highly selective reaction that leads to production of aldehyde groups [44], successful periodate oxidation is dependent on optimal oxidation conditions, which include concentration, time and temperature of reaction [44,45,48,49].

Reports have shown that during periodate oxidation, the aldehyde content in cellulose (cotton) monotonically increases with increase in reaction time [50,51]. Thus, as the reaction time increases, more hydroxyl groups are converted to the aldehyde C = O group [52]. In addition, reports have shown that longer reaction times led to the disappearance of the stretching vibration of the carbonyl group, which might be due to a result of side products forming with increased reaction time [52]. Time as a determinant in periodate oxidation cannot be taken in isolation. Report shows that the reaction time is dependent on three other factors [53], the reaction pH being the most influential factor, temperature, and ratio of the material being conjugated, relative to the oxidant. [49]. In this work, periodate oxidation was carried out for different time periods. Our data suggests an optimum oxidation time of 9 h.

With regard to pH, periodate oxidation of cellulose is reported to be more efficient in the presence of acidic conditions [45,48,54]. However, reaction in strong acid media is discouraged as it leads to low-product yields owing to the physical and chemical degradation of the oxidised cotton swab [49,55]. Hence, selecting a proper pH value is critical to aldehyde formation as the most influential factor [49]. The pH of 5.2 used in our study falls within the reported pH range of 2.0–6.0 for efficient periodate oxidation. It has been reported that aldehyde content decreases with increasing pH values because selective oxidation of the hydroxyl groups at the C2 and C3 positions by NaIO_4_ in cellulose requires the use of H^+^ as a catalyst [56]. Thus, increasing the pH results in an exponential decrease in the H^+^ concentration, leading to a decrease in the efficiency of the oxidation reaction and a reduction in the aldehyde produced [49].

As observed in this study, increase in concentration of periodate increases the rate of oxidation (Supplementary Data S1–S4). This corroborates previous reports on periodate oxidation of cotton [44,45,48], but the strength of the oxidised cotton decreased at high concentrations of NaIO_4_ over a duration greater than 10 h. This is adduced to the breakdown in structure of cellulose as a result of oxidation, since oxidation by sodium periodate breaks the crystalline structure of cellulose in the cotton [44].

In the literature, a temperature range from room temperature (25 °C) to 55 °C has been reported for periodate oxidation [48,54,56]. The optimum temperature of 35 °C reported in our study is in line with recent data [54]. Given that the oxidation reaction of sodium periodate is endothermic [54] an increasing reaction temperature will result in a higher aldehyde at the initial stage of the reaction. Zhang and colleagues [54] reported that sodium periodate will decompose easily at high temperatures, resulting in oxidant loss. Further, they reported that the aldehyde content was lower at an oxidation reaction temperature of 45 °C than at the oxidation reaction temperatures of 30 °C or 35 °C. In their study, the aldehyde content reached a maximum at 35 °C but their product yield reached a maximum at 30 °C, hence, the temperature of 30 °C was chosen as the optimum synthesis temperature in their experiment. Interestingly, our study reports that aldehyde content reaches a maximum at 35 °C and we chose this as our optimum temperature since our interest is in the aldehyde content, needed for the lactoferrin immobilisation for rotavirus kit development.

Our developed immunoassay is for diagnosis of rotavirus, a double stranded RNA virus, the major cause of diarrhoea morbidity and mortality in children. To the best of our knowledge, no previous studies have reported the optimisation of periodate oxidation conditions for aldehyde production in the development of viral nanoparticle-based immunoassay. Our study also reports for the first time, the suitability of such assay as a bedside tool in rotavirus diagnosis among diarrhoeic children.

A few studies have reported rotavirus detection using nanoparticles [57,58,59] in immunochromatographic test strip [57] and surface-enhanced Raman scattering [58,59] but the fabrication process and the detection procedure of these methods are complicated. Our method has the advantage of visual detection platform without the use of specialised equipment and a short detection time. The result was obtained in less than 30 min when the assay was used for RV detection in the diarrhoeic and control samples, showing a fast RV diagnosis result that could be performed per sample, revealing its suitability as a bedside rotavirus diagnostic kit. This is important since accurate and timely identification of pathogens is necessary in appropriate treatment and infection control of rotavirus [60].

Our developed immunoassay is suitable for detecting rotavirus gastrointestinal infection in children, the most vulnerable group to rotavirus infection. Although there are various commercial rotavirus rapid test strips with good specificity when compared with molecular methods, the sensitivity is usually lower [61,62,63]. Malla et al., 2024 compared the performance of a rapid lateral flow agglutination assay with PCR and discovered a diagnostic sensitivity and specificity of 74.54% and 98.44% in comparison with RT-PCR [61], while a sensitivity of 75.5% and specificity of 98.2% have also being reported [62]. Generally, the reported sensitivity and specificity of rapid kits range between 52 and 95.08%, respectively [61,62,63]. ELISA is the endorsed World Health Organization (WHO) standard method for rotavirus antigen detection in stool samples [61,64] and the method has been reported to have better performance than rapid kits [61,63]. We therefore decided to compare our developed assay with ELISA rather than a rapid kit, since ELISA performs better and it is the WHO-endorsed method. In addition, the use of cotton swab in our assay compared to other rapid tests makes sample collection easier, even if the child is in bed and uses diapers. The cotton swab serves the dual purpose of facilitating sample collection and a detection platform.

The performance of our nanoparticle-based immunoassay, in comparison with ELISA in detecting rotavirus in diarrhoea children, using PCR as gold standard showed that the nano-assay has a better performance and compares well with the molecular method. The performance of ELISA against the molecular method as the gold standard showed that the sensitivity of the method in true RV detection is low at 60% but has a better specificity with precision of 88% in contrast to our developed kit which showed a better performance at 88% and 94% sensitivity and specificity, respectively. A previous report had also shown that a Latex agglutination (LA) test performed better that ELISA in rotavirus detection when PCR was used as gold standard [65].

The performance of ELISA in our study in terms of sensitivity and specificity aligned closely to reports by Hoque et al. (2019) [66] and Barsoum (2020) [67], although lower values [68] and higher values have also been published [27,69]. A 53% PPV indicated that about half of the positive results were false positives, highlighting the risk of over-diagnosis using ELISA which could be due to cross-reactivity, instrumental calibration or operational error, which brings about the possibility of ELISA diagnosing an infection in a person even if they are asymptomatic or have had an infection in the past [25]. On the other hand, the 88% NPV indicated that ELISA was reliable in ruling out rotavirus infection from genuinely negative samples. Reports have shown that ELISA NPV is usually higher that PPV [65,69] as observed in our study.

The high accuracy and reliability demonstrated by the nanoparticle-based diagnostic immunoassay in detecting RV infection makes it a promising tool for rapid diagnosis and timely intervention. The high SS and SP shows that it compares well with molecular method as the gold standard and will find relevance as a suitable alternative to molecular methods especially in regions where molecular method is not affordable.

We observed that using the nanoassay, there were eight samples with false positive results when compared with the PCR (qRT-PCR) used as gold standard. In resolving this, only samples with high viral concentration (Concentration ≥ cut-off (5 × 10^5^) = symptomatic RVA), hence low C_t_ score values were considered as positive since previous reports have shown that they are associated with symptomatic RVA cases [39]. It was observed that the 8 samples had high C_t_ score values of between 30 and 34 (low viral loads), and hence were from asymptomatic cases. This showed the inability of our assay to differentiate rotavirus antigen positive samples with very low viral concentration, and hence the asymptomatic cases from the symptomatic cases. Despite this shortcoming, the performance of our immunoassay is comparable to or even surpasses that of other reported diagnostic methods [70,71,72], including ELISA in this study. Moreso, the intensity of the colour change for these asymptomatic cases was not as bright as those symptomatic cases with high viral load.

The data generated from the diarrhoeic and control groups showed that the intensity of the colour change is proportional to the viral concentrations for the positive samples when compared to the qPCR. This defines the critical next steps in pursuit of a robust improved method which will be quantitative, and can differentiate asymptomatic from symptomatic rotavirus infection, since all the false positive samples actually have very high C_t_ score values and not too bright colour change. Interestingly, our assay showed that all the control cases were RV negative. However, five positive samples were also falsely reported as negative, despite having low C_t_ score values.

The limitation of the study is the fact that the immunoassay is not quantitative. Similarly, some studies have reported the use of nanoparticles for biosensing in the development of bacterial or viral diagnostic kits [31,32,33,34]. Nevertheless, our use of nanoparticles for rotavirus as well as its evaluation in clinical samples is novel. Also, our assay was not tested for other viruses, nor was the limit of detection (LOD) determined. The monoclonal antibody ensures specificity for this qualitative assay. The non-determination of the LOD does not invalidate the results which were confirmed by the qPCR data. The LOD and other quantification are proposed to be evaluated in subsequent optimisation to achieve a quantitative assay. In addition, the differences in colour intensity between symptomatic and asymptomatic cases when compared with C_t_ score values support the possibility of assay standardisation to a quantitative nanokit with further studies, thereby resolving this limitation. The overall strength and relevance of our study is in the fact that no previous studies reported nanoparticle-based rotavirus immunoassay detection either as a proof of concept or in real cases among diarrhoea and control subjects, nor was the optimal condition for periodate oxidation studied, making our study unique and reproducible.

## 5. Conclusions

This study reports the successful development of a rapid, cheap, sensitive, simple and equipment free nanoparticle-based rotavirus diagnostic kit, it describes the optimal condition for NaIO_4_ oxidation of cotton swab for aldehyde production and shows the performance of the developed kit in RV detection infection among diarrhoea and control groups. The developed assay will be suitable for routine diagnosis of RV infection in settings where molecular method is not affordable.

## Figures and Tables

**Table 1:** Comparison of RV detection by ELISA, the developed nanoparticle-based immunoassay and PCR in Diarrhoeic and control Group.

Result	ELISA			Nanoparticle Based Kit			qRT-PCR		
	Diarrhoeic Group	Control	Total	Diarrhoeic Group	Control	Total	Diarrhoeic Group	Control	Total
Positive	41 (34%)	6 (9%)	47 (25%)	45 (37%)	0 (0%)	45	43 (36%)	1 (2%)	44 (24%)
Negative	80 (66%)	59 (91%)	139 (75%)	76 (63%)	65 (100%)	141 (76%)	78 (65%)	64 (99%)	142 (76%)
Total	121	65	186	121	65	186	121	65	186

**Table 2:** (**a**,**b**) The true positives and Negatives RV detection by the developed nanoparticle-based immunoassay in comparison with qPCR in Diarrhoeic and control Group.

**(a)**				
	**Nanoparticle-Based Kit**	**Cases**	**Control**	**PCR**
True +ve	37	37	0	44
True −ve	136	72	64	142
False +ve	8	8	0	
False −ve	5	4	1	
	186	121	65	
**(b)**				
	**ELISA**	**Cases**	**Controls**	**PCR**
True +ve	25	25	0	44
True −ve	122	64	58	142
False +ve	22	16	6	
False −ve	17	16	1	
	186	121	65	

**Table 3:** Comparison of ELISA and Nanoparticle-based immunoassay performance using qPCR as gold standard.

Assay	Sensitivity (95% Cl)	Specificity (95% Cl)	PPV (95% Cl)	NPV (95%Cl)
ELISA	0.60 (0.43–0.74)	0.84 (0.78–0.90)	0.53 (0.38–0.68)	0.88 (0.81–0.93)
Nanoparticle Kit	0.88 (0.74–0.96)	0.94 (0.89–0.98)	0.82 (0.68–0.92)	0.96 (0.92–0.99)

## Data Availability

Data and figures generated or analysed during this study are included in the manuscript, while Supplementary Data S1–S5 is submitted as an attachment.

## Supplementary Materials

The following supporting information can be downloaded at: https://www.mdpi.com/article/10.3390/mps8040081/s1, Supplementary Data S1: FTIR spectra of oxidised cotton swab at 3 h using varying concentration of NaIO_4_ in 0.1 M sodium acetate buffer; Supplementary Data S2: FTIR spectra of oxidised cotton swab at 6 h using varying concentrations of NaIO_4_ in 0.1 M sodium acetate buffer; Supplementary Data S3: FTIR spectra of oxidised cotton swab at 9 h using varying concentrations of NaIO_4_ in 0.1 M sodium acetate buffer; Supplementary Data S4: FTIR spectra of oxidised cotton swab at 24 h using varying concentrations of NaIO_4_ in 0.1 M sodium acetate buffer; Supplementary Data S5: FTIR spectra of oxidised cotton swab before and after lactoferrin coupling.

## Abbreviations

The following abbreviations are used in this manuscript:

RVA	Group A rotavirus (RVA)
LF	Lactoferrin
RV	Rotavirus
FT-IR	Fourier-Transform Infrared Spectroscopy
LA	Latex agglutination
ELISA	Enzyme Linked Immunosorbent Assay
NaIO_4_	Sodium periodate
PBS	Phosphate-buffered saline
EDC	1-ethyl-3-(3-dimethylaminopropy)carbodiimide hydrochloride
BSA	Bovine serum albumin
qRT-PCR	Quantitative Reverse Transcription PCR
MS2	2-(N-morpholino) ethanesulfonic acid
NHS	N-hydroxy succinimide
Ag	Antigen
PCR	Polymerase chain reaction
