# Supplementary material for: Development and Evaluation of a Nanoparticle-Based Immunoassay for Rotavirus Detection: A Suitable Alternative to ELISA and PCR in Low-Income Setting
Source: Methods Protoc. 2025 Jul 17;8(4):81. doi: 10.3390/mps8040081 (PMC12286212; doi:10.3390/mps8040081)
Supplement: Supplementary file 1 [file mps-08-00081-s001.zip › mps-3676443-supplementary.pdf]

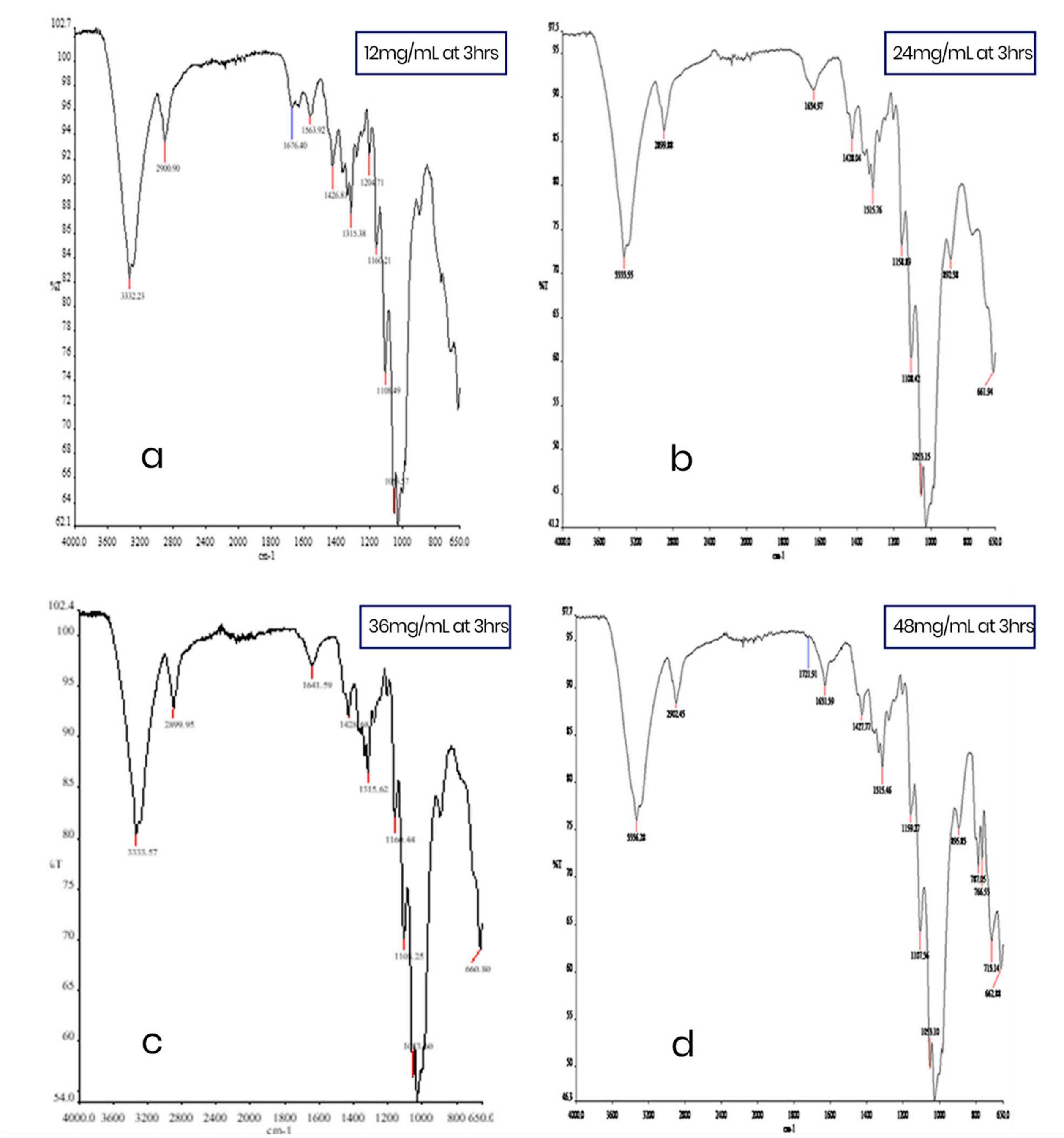

Data S1: FTIR spectra of oxidised cotton swab at 3h using 12mg/mL, 24mg/mL, 36mg/mL and 48mg/mL $NaIO_4$ in 0.1M sodium acetate buffer

In Data S1 involving oxidation for 3h, no aldehyde peak was seen on swabs oxidised for 3 hours using 12-36mg/mL (a-c) NaIO4, but a small peak was seen at a higher concentration of 48mg/mL (d).

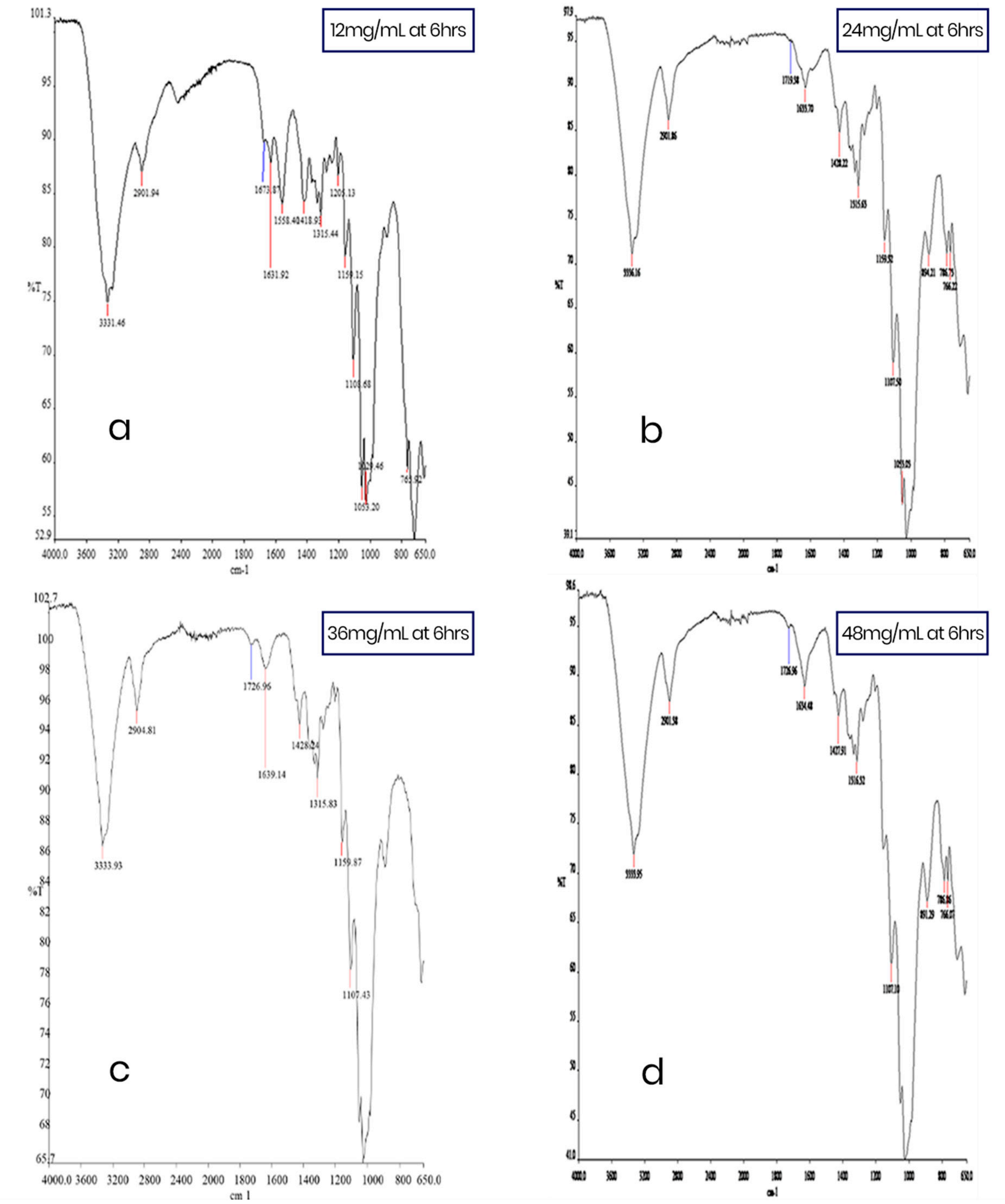

Data S2: FTIR spectra of oxidised cotton swab at 6h using 12mg/mL, 24mg/mL, 36mg/mL and 48mg/mL $NaIO_4$ in 0.1M sodium acetate buffer (a-d).

For the 6-hour experiments (Data S2), a similar trend was observed, however, with the use of 36mg/mL (c) and 48mg/mL (d), minor aldehyde peaks could be seen of the FT-IR spectra.

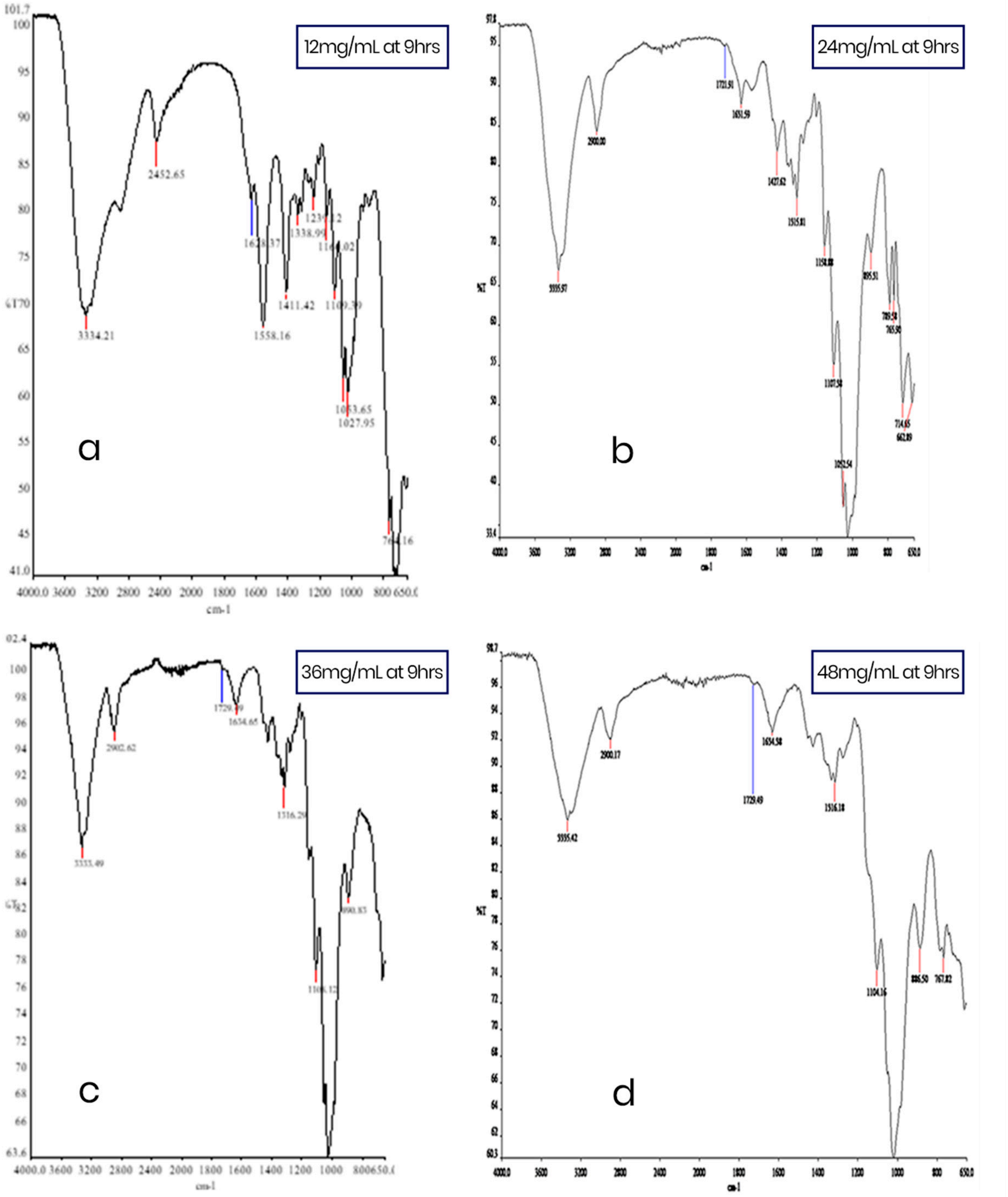

Data S3: FTIR spectra of oxidised cotton swab at 9h using 12mg/mL, 24mg/mL, 36mg/mL and 48mg/mL $NaIO_4$ in 0.1M sodium acetate buffer (a-d).

For the 9-hour experiments (Data S3), no aldehyde peak was observed for the reaction using 12mg/mL (a) but minor aldehyde peaks could be seen using 24mg/mL (b) and 36mg/mL (c). A distinct peak at 1729 cm-1 was observed using 48mg/mL NaIO4 (d).

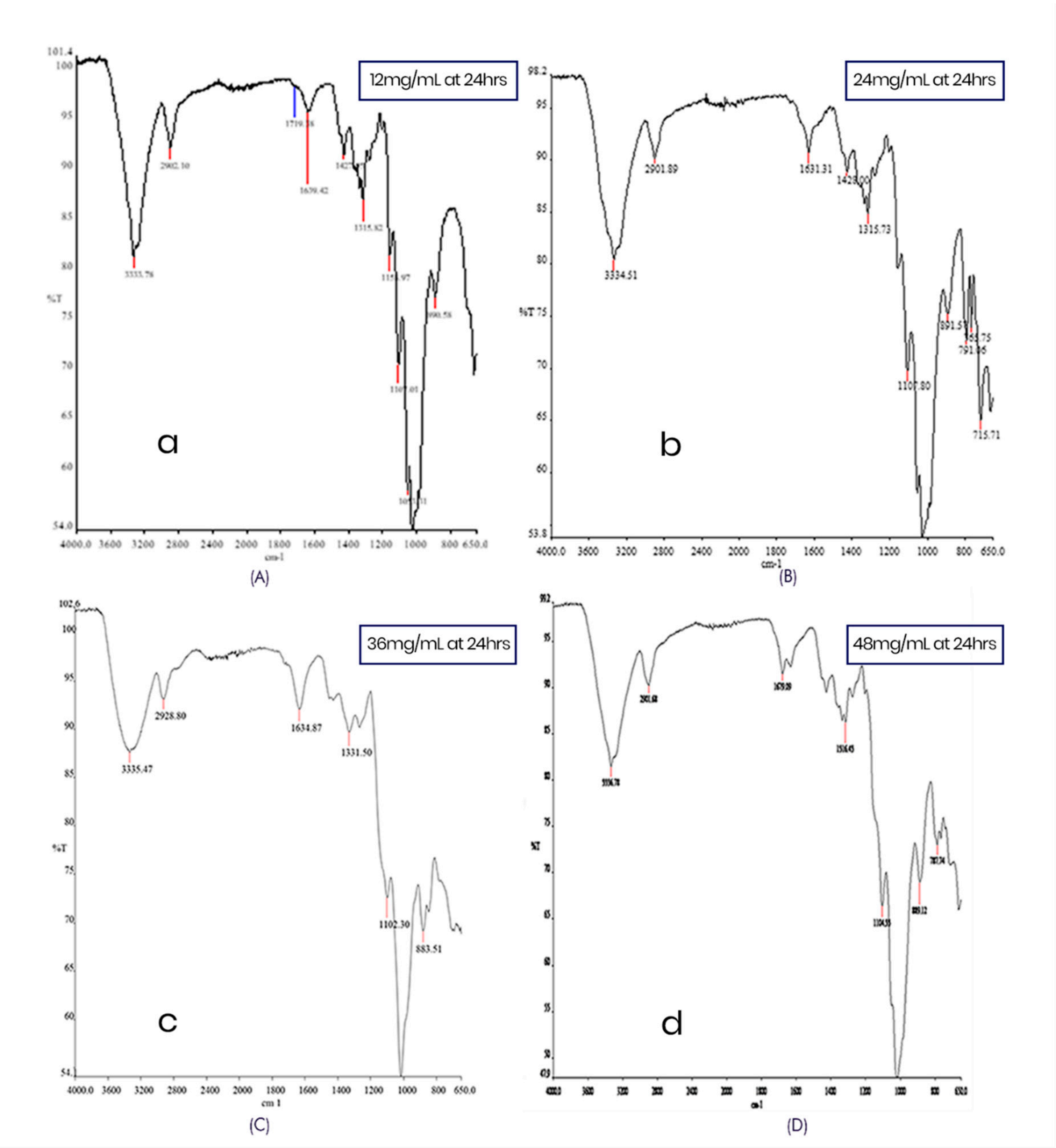

Data S4: FTIR spectra of oxidised cotton swab at 24 h using 12mg/mL, 24mg/mL, 36mg/mL and 48mg/mL $NaIO_4$ in 0.1M sodium acetate buffer (a-d).

For the 24-hour reactions (Data S4), a minor aldehyde peaks were observed at the lowest concentration and was not evident at the other concentrations of NaIO4. Thus, our data suggest that as the NaIO4 concentration increased, there was a corresponding increase in the degree of cotton swab oxidation with an increase in time, up to an optimum, after which the oxidation reaction was impaired.

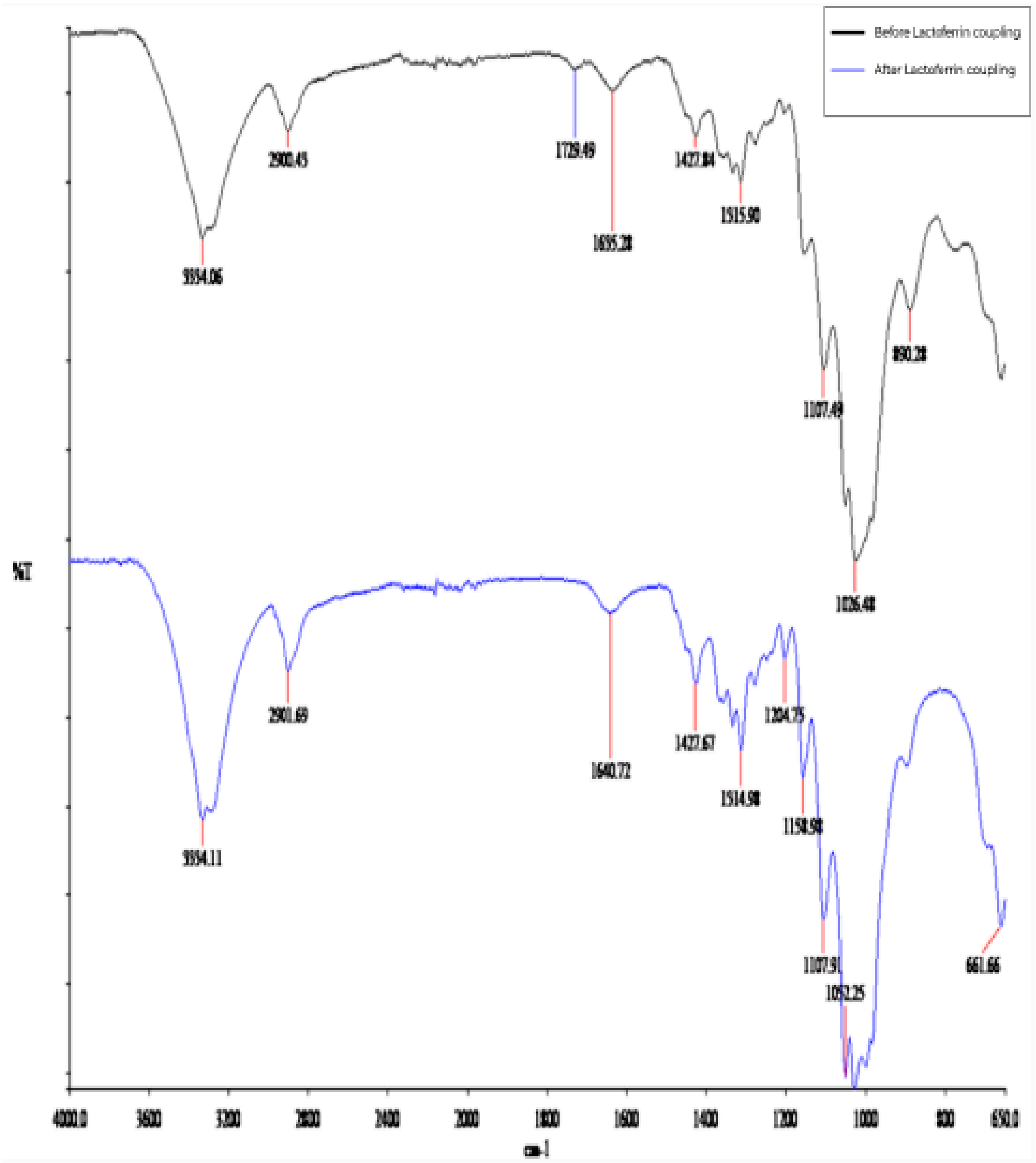

Data S5**.** FTIR spectra of oxidised cotton swab before lactoferrin coupling (top)and after lactoferrin coupling (bottom) showing the disappearance of the aldehyde peaks.
